# Altered spontaneous brain activity in Down syndrome and its relation with cognitive outcome

**DOI:** 10.1038/s41598-022-19627-1

**Published:** 2022-09-14

**Authors:** Cristina Cañete-Massé, Maria Carbó-Carreté, Maribel Peró-Cebollero, Shi-Xian Cui, Chao-Gan Yan, Joan Guàrdia-Olmos

**Affiliations:** 1 Department of Social Psychology and Quantitative Psychology, Faculty of Psychology, Universitat de Barcelona, Barcelona, Spain; 2 UB Institute of Complex Systems, Universitat de Barcelona, Barcelona, Spain; 3 Serra Hunter Fellow, Department of Cognition, Development and Educational Psychology, Faculty of Psychology, Universitat de Barcelona, Barcelona, Spain; 4 Institute of Neuroscience, Universitat de Barcelona, Barcelona, Spain; 5 CAS Key Laboratory of Behavioural Science, Institute of Psychology, Beijing, China; 6 International Big-Data Center for Depression Research, Chinese Academy of Sciences, Beijing, China; 7 Magnetic Resonance Imaging Research Center, Institute of Psychology, Chinese Academy of Sciences, Beijing, China; 8 Sino-Danish College, Sino-Danish Center for Education and Research, University of Chinese Academy of Sciences, Beijing, China

**Keywords:** Neuroscience, Cognitive neuroscience

## Abstract

Although Down syndrome (DS) is the most common genetic cause of neurodevelopmental delay, few neuroimaging studies have explored this population. This investigation aimed to study whole-brain resting-state spontaneous brain activity using fractional amplitude of low-frequency fluctuation (fALFF) and regional homogeneity (ReHo) strategies to find differences in spontaneous brain activity among young people with DS and controls and to correlate these results with cognitive outcomes. The sample comprised 18 persons with DS (age mean = 28.67, standard deviation = 4.18) and 18 controls (age mean = 28.56, standard deviation = 4.26). fALFF and ReHo analyses were performed, and the results were correlated with other cognitive variables also collected (KBIT-2 and verbal fluency test). Increased activity was found in DS using fALFF in areas involving the frontal and temporal lobes and left cerebellum anterior lobe. Decreased activity in DS was found in the left parietal and occipital lobe, the left limbic lobe and the left cerebellum posterior lobe. ReHo analysis showed increased activity in certain DS areas of the left frontal lobe and left rectus, as well as the inferior temporal lobe. The areas with decreased activity in the DS participants were regions of the frontal lobe and the right limbic lobe. Altered fALFF and ReHo were found in the DS population, and this alteration could predict the cognitive abilities of the participants. To our knowledge, this is the first study to explore regional spontaneous brain activity in a population with DS. Moreover, this study suggests the possibility of using fALFF and ReHo as biomarkers of cognitive function, which is highly important given the difficulties in cognitively evaluating this population to assess dementia. More research is needed, however, to demonstrate its utility.

## Introduction

Down syndrome (DS) is the most common genetic cause of neurodevelopmental delay and affects one out of 700 live births^1^. Although the life expectancy of people with DS has increased dramatically^2,3^, age-related comorbidities in this group have appeared, specifically Alzheimer's disease (AD)^4^. In the last few years, new quantitative approaches using brain magnetic resonance imaging (MRI) have characterized brain development in diseased and healthy populations^5^. In the field of DS, although clinical and genetic characteristics have been described, few neuroimaging studies have been performed^6^.

Regarding structural MRI, brain abnormalities have been demonstrated in the DS population. Koenig et al.^7^ demonstrated differences in the hippocampus in the DS group compared with the non-DS group. Similarly, Beacher et al.^8^ demonstrated that the frontal, temporal, and parietal lobes show more significant age-related reduction in DS than in the general population. Additionally, white matter integrity has been found to be decreased in DS and correlates with cognitive dysfunction^9,10^. Benjanin et al.^11^ found an association between cognitive decline and the APOE e4 allele, the earlier loss of cortical metabolism and hippocampal volume; these results were congruent with previous studies on brain volume loss^12–14^. APOE e4 is the most established genetic risk factor for sporadic AD and has been related to earlier symptoms in the general population and in DS^11^.

Less is known about functional MRI (*f*MRI) in DS; nevertheless, a systematic review^6^ highlighted the importance of studying resting-state functional MRI (rs-*f*MRI) in this population. The Rs-*f*MRI paradigm captures intrinsic functional spontaneous brain activity and allows the evaluation of the brain baseline function and has been used to elucidate differences between diseased populations and control participants^15^. This paradigm has been suggested as a suitable biomarker for abnormal brain function and predicting later adverse neurodevelopment in DS^16^. The majority of studies using rs-*f*MRI in this population have focused on specific areas or predetermined networks, such as the default mode network^17–19^ (DMN) or the hippocampus^7^. However, few studies have focused on the whole brain^20^.

Unclear findings in functional connectivity (FC) have been found in this field for various reasons. First, there is heterogeneity in the age of the participants. In this sense, it is crucial to consider that AD neuropathology is universal by 40 years of age in people with DS^21,22^, and therefore, significant changes in the brain can occur in this population in an interval of very few years. It has been demonstrated that the DS population exhibits a β-amyloid (Aβ) burden prior to dementia diagnosis, and this deposition can begin in the late teens^23–25^. Therefore, it is vital to focus investigations on young DS participants who have not yet developed the neuropathology of AD. These studies can help to elucidate the abnormalities of the DS phenotype and aid in finding biomarkers of neurodegeneration^16^. Second, the use of predetermined seeds or regions of interest limits the comparison of results between studies. Third, methodological decisions, for instance, different head motion corrections or approaches of analyses, can also limit the generalization of the findings^26^. Finally, the limited number of studies and the small sample size typically used have also limited the reliability of these findings^27,28^.

Despite these limitations, studies have demonstrated altered FC in this population. Increased brain synchrony was found between distributed brain regions, including grey matter, visual-frontoparietal regions, somatomotor regions, and different regions of the DMN and frontal lobes^29–31^. The DMN is related to high structural and FC while a person is at rest and has been demonstrated to be disrupted in several diseases^32^, such as DS. Results indicating abnormalities in DS through the DMN were found^17–19^: first, a disrupted connectivity between posterior brain regions, and second, hyperconnectivity and hypoconnectivity, including different subregions of the DMN. Finally, they found weaker strength in frontal regions, consistent with other studies^29^. Recently, Csumitta et al.^20^ found increased whole-brain FC in DS.

Some studies have also tried to link the abnormalities with different neurocognitive impairments typically found in DS. In this sense, the cognitive domains that are particularly impaired in individuals with DS, apart from an intelligence quotient (IQ) usually ranging from 30 to 70, include language (particularly expressive language), memory, executive function and motor coordination^33^. Anderson et al.^29^ found an inverse relationship between network synchrony performance and IQ in the DS group, whereas Vega et al.^31^ found no relationship between the abnormalities in network connectivity and IQ. Pujol et al.^30^ evaluated whether the differences found in FC could be related to communication skills and found direct relationships between FC in the ventralial frontal cortex, amygdala and communication skills. They also found indirect relationships between communication skills and decreased FC in the left posterior insula and right sensorimotor cortex. These results are in accordance with Csumitta et al.^20^, who demonstrated that differences found in connectivity in DS could be related to verbal abilities.

Functional neuroimaging findings until now have suggested alterations in connectivity in DS. However, the vast majority of studies are linked with finding differences between controls and DS in FC. However, FC only depicts the relationship between two or more regions and thus does not provide information on which exact single region is abnormal within networks. In contrast, regional spontaneous brain activity analysis may provide this helpful information^34^ and thus could help disentangle differences in regional activity^35^.

The amplitude of low-frequency fluctuations (ALFF) and regional homogeneity (ReHo) are data-driven methods that reveal different regional characteristics of rs-fMRI data and are useful when the studies in the population are very limited; therefore, there are no hypotheses on specific regions. ALFF and ReHo have recently been used in many psychiatric diseases^36–38^, neurodevelopmental disorders^39,40^, and dementias^41,42^; however, to our knowledge, these methods have not yet been applied in the DS population, and both methods seem promising for detecting regional signal changes in spontaneous activity. Both tools have been suggested as potential biomarkers for tracing changes in the brain while it develops, as well as in relation to behaviours and diseases, as both techniques present high temporal stability^43,44^.

On the one hand, ALFF measures the strength of the regional intensity of spontaneous fluctuations in the BOLD signal^45^ but has often been criticized because it could be sensitive to physiological noise. Therefore, Zou et al.^34^ suggested fractional amplitude of low-frequency fluctuations (fALFF), which enhances the sensitivity and specificity of spontaneous brain activity detection. On the other hand, ReHo estimates the temporal homogeneity of the signal between a given voxel and neighbouring voxels. Basically, ReHo estimates local neural activity. Both approaches are complementary, while fALFF is focused on measuring local spontaneous activity, and ReHo estimates regional abnormalities^34^. Thus, combining ReHo and fALFF to assess spontaneous brain activity among the DS population could provide more information about brain function in people who present an intellectual disability (ID).

In addition to the interesting use of ReHo and fALFF to explore the brain mechanism of DS, it could be interesting to examine the relationship with other external, as it has been done in other populations. Li et al.^40^ studied ALFF and ReHo with a sample of individuals with low-functioning autism spectrum disorder (ASD) and found increased ReHo and ALFF in different brain regions. However, no correlation was found with clinical symptoms in the ASD group. Lee & Hsiesh^46^ studied fALFF and ReHo in a healthy population and showed a significant relationship with cognitive outcome using a stop-signal task and found negative correlations with different areas using both approximations. In the AD and mild cognitive impairment (MCI) field, Yang et al.^47,48^ found significant correlations between fALFF and ReHo values and neuropsychological assessment in both populations. More interestingly, Li et al.^49^ studied the relationship between fALFF and ReHo and amyloid-β accumulation in a sample with subjective cognitive decline (SCD) and found higher ReHO in the precuneus and superior parietal areas in amyloid-positive patients. Lu et al.^50^ also found significant correlations between fALFF and executive function in a sample of childhood trauma in young adults. Fryer et al.^51^ also found significant correlations between fALFF measures and cognitive function in schizophrenia. Consequently, both fALFF and ReHo seem to be promising techniques related to cognitive or clinical symptoms.

The present paper aimed to study the whole-brain resting state using fALFF and ReHo strategies to find differences in spontaneous brain activity among young people with DS and controls. We hypothesized that significant differences would be found between both groups, as seen in other rs-fMRI studies. As both techniques are data-driven methods and have never been used in DS, there was no need for a hypothesis. However, both techniques have been found to be highly related to FC^52^; therefore, the study of regional activity could elucidate already discovered FC abnormalities. Consequently, we expected to find differences in the frontal lobe and in the DMN^17–19,30^, following other resting-state studies in this population. Moreover, as there are cognitively impaired domains in DS^33^, we expected that the neural correlates associated with these functions would be altered in spontaneous activity. For instance, the temporal and frontal lobes seem to play an important role in language and executive functions^53^, as well as the frontoparietal brain regions in memory^54^. In addition, we expected the differences in fALFF and ReHo between DS and controls to be related to cognitive outcomes, as in other studies^20,29^.

## Methods

### Participants

The sample was comprised of 20 persons with DS and 20 non-DS controls matched by chronological age and gender. However, due to excessive movement in the *f*MRI registration, the final sample was comprised of 18 persons with DS (5 females, mean age = 28.7, standard deviation (SD) of age = 4.18) and 18 controls (mean of age = 28.56, SD of age = 4.26). In both groups, the same protocol was applied. The recruitment of the DS group was conducted through different centres attending people with ID in Catalonia, Spain. The recruitment of the control participants was made from the community through advertisements. Regarding the inclusion criteria of the participants, the DS group had to be between 16 and 35 years old, and all of them had to have a diagnosis of DS. The participants with DS were excluded if: other comorbid diagnoses implying cognitive dysfunction were present apart from the DS diagnosis itself, the legal guardian’s consent could not be obtained, and the person with DS had medication that could affect cognitive function (for example, anxiety medication). Control participants had to be matched by gender and age (± 2 years) with DS participants. They were excluded if they had any psychiatric diagnoses or other disorders affecting cognitive function. For both groups, if excessive movement was present in the registration of the *f*MRI sequences, information for that participant was discarded. IQ was estimated for both groups with the Kaufman Brief Intelligence Test, Second Edition (KBIT-2)^55^. In the DS group, the mean KBIT-2 Full Scale IQ score was 43.94 ± 6.2 (range 40–66). The demographic information of the sample appears in Table 1.

**Table 1:** Participant characteristics.

	DS (mean; SD)	Controls (mean; SD)	Test (*p value*)
Age (years)	28.67 (4.18)	28.56 (4.26)	*Z* = − 0.03 (*p* = 0.975)
Gender (% male)	72.22%	72.22%	
Head motion	0.19 mm (0.10)	0.08 mm (0.03)	*Z* = − 4.46 (*p* < 0.001)
Phonological verbal fluency	4.28 (3.30)	18.11 (5.41)	*Z* = − 5.14 (*p* < 0.001)
Semantic verbal fluency	17.50 (10.86)	51.16 (12.05)	*Z* = − 4.84 (*p* < 0.001)
KBIT-2 Vocabulary (DS group, n = 17)	25.41 (12.23)	71.72 (4.10)	*Z* = − 5.06 (*p* < 0.001)
KBIT-2 Matrices KBIT-2 (DS group, n = 17)	13.17 (5.44)	39.33 (3.34)	*Z* = − 5.06 (*p* < 0.001)
KBIT-2 raw total (DS group, n = 17)	96.88 818.18	223.5 (13.73)	*Z* = − 5.05 (*p* < 0.001)
KBIT-2 Full IQ standardized (DS group, n = 17)	43.94 (6.23)	111.05 (7.83)	*Z* = − 5.31 (*p* < 0.001)

*Z* Z score linked to the Mann–Whitney test, *SD* standard deviation.

### Procedure

The Bioethical Committee of the Universitat de Barcelona approved the project (03/16/2017), and all methods were performed in accordance with the relevant guidelines and regulations. For minors and the participants with DS, informed consent of the guardians in legal charge of every person with DS was obtained. Informed consent was also acquired from all DS and control participants.

As the data of this project belong to a more extensive protocol, more data than the one presented here were registered. In this sense, the data used in this study only comprised the following: a usual sociodemographic questionnaire and a checklist for the MRI scanner. A cognitive test, KBIT-2^55^, and a simple verbal fluency test were performed. The KBIT-2 test has been used to evaluate people with DS and controls in multiple studies^20,29^ and was chosen because of its ability to ensure items were age-appropriate, as the age rank for the administration is sufficiently large. Moreover, it evaluates verbal and nonverbal IQ. The measures used were raw scores in matrices (nonverbal), vocabulary (verbal) and total IQ. Moreover, the standardized score for total IQ was also used. It is important to mention that data were missing for one subject with DS to whom the KBIT-2 was not administered. Hence, the total sample with the KBIT-2 test was n = 35.

Finally, regarding the verbal fluency test, three measures were performed: (a) phonological verbal fluency: evaluated by the number of words produced beginning with the letter “p” during 1 min; (b) semantic verbal fluency: evaluated by the number of words produced during 1 min related to things that can be bought in a supermarket; and (c) semantic verbal fluency: evaluated by the number of words produced during 1 min related to the names of colours. The total score for semantic verbal fluency was estimated by adding the (b) and (c) scores.

Participants were evaluated in two sessions, and the sequence was the same for the DS and control participants. Questionnaires were administered, and images were acquired in the first session. The second session was dedicated to another part of the general research design.

### MRI acquisition

Brain imaging was performed in a Philips Ingenia 3 MRI scanner T system located in the Fundació Pasqual Maragall in Barcelona, Spain. All participants underwent a *f*MRI recording sequence: T1, T2, Flair, and resting state. Regarding the resting-state registration, participants with DS only underwent a total of 6 min in the MRI scanner. Participants with DS underwent a short training to improve their familiarization with the scan and acclimate to the noise and environment. The control participants underwent a sequence of 10 min, but for this study, only the first 220 volumes of registration were used to guarantee a possible comparison between both groups. All participants were told to try to stay quiet without movement. Moreover, they should remain awake and with their eyes opened and fixed on a cross symbol on the screen. Participants could choose music to hear during all recordings except in the resting-state scan. Although individuals in the eyes-closed condition are more likely to become drowsy and fall asleep^56^, none of the subjects included fell asleep during scanning, as self-reported after scanning. A T1-weighted turbo field echo (TFE) structural image was obtained for each subject with a 3-dimensional protocol (repetition time (TR) = 2300 ms, echo time (TE) = 2980 ms, 240 slices, and field of view (FOV) = 240 × 240 × 170). The image acquisition was in the sagittal plane. For the functional images, a T2*-weighted (BOLD) image was obtained (TR = 1750 ms, TE = 30 ms, FOV = 230 × 230 × 160, and voxel size = 3 × 3 × 3 mm, 46 slices). The image acquisition was in the transverse plane.

### Data preprocessing

Image preprocessing was performed using the Data Processing Assistant for Resting-State fMRI^57^ (DPARSF; http://rfmri.org/DPARSF). Basically, it is based on MATLAB, SPM12 (http://www.fil.ion.ucl.ac.uk/spm) and DPABI^58^.

The first 10 functional images were removed to allow magnetization equilibration and to allow participants to adapt to the scanner. Then, a correction was made for the remaining functional images for slice acquisition timing difference and head motion. Nuisance signals were regressed out, including white matter signals, cerebrospinal fluid signals, linear trends and signals associated with the 24 Friston head-motion parameters^59^. The derived functional images were coregistered with the corresponding structural images, which were segmented and normalized to Montreal Neurological Institute (MNI) space using diffeomorphic anatomical registration through exponentiated lie algebra (DARTEL). The functional images were normalized to MNI space with warped parameters and resampled to 3 mm cubic voxels. For the ReHo analysis, the normalized functional images were then bandpass filtered (0.01–0.1 Hz). As DS is a specific population that can present excessive movement, the criterion used to exclude subjects in the sample was that the participants could not exceed the mean of the group plus 2 SD^60^, estimated with Jenkinson’s framewise displacement^61^ (FD) owing to its consideration of voxelwise differences in motion in its derivation^60^. Overall, two persons with DS were excluded, and the final sample included 18 people with DS and 18 controls. Both groups differed in movement (*Z* = − 4.46, *p* < 0.001, $\overline{x }$_DS_ = 0.19 (SD = 0.10); $\overline{x }$_C_ = 0.08 (SD = 0.03)), presenting more movement in the DS group. The DS group presented a total of 1040 (out of 3780) volumes exceeding 0.2 mm in Jenkinson FD, whereas the control participants presented a total of 172 volumes exceeding 0.2 mm in Jenkinson FD. Therefore, scrubbing regression was performed^60,62,63^ in the final step of the preprocessing to control the movement exceeding 0.2 Jenkinson’s FD. We further addressed the residual effects of motion in group analyses by including mean FD derived from Jenkinson’s FD as a nuisance covariate^52^.

### Calculation of fALFF and ReHo

The estimation of fALFF and ReHo values was performed using DPABI^58^. To estimate ALFF, spatial smoothing was performed with a 4 mm full width at half maximum (FWHM) Gaussian Kernell. To compute the power spectrum, the time series of each voxel was transformed to the frequency domain via fast Fourier transform (FFT). This power spectrum, which has a frequency range of 0–0.25 Hz, was square-rooted at each frequency and then averaged across 0.01–0.08 Hz at each voxel, which was taken as ALFF. To obtain fALFF, the ALFF values were divided by the whole frequency range observed in the signal^34^ (0–0.25 Hz).

Regarding the ReHo estimation, Kendall’s concordance coefficient (KCC) of the time series of all voxels and their neighbours was calculated^35^. All ReHo maps were smoothed with a Gaussian kernel of four mm FWHM. Finally, individual fALFF and ReHo maps were standardized into z score maps by subtracting the mean and dividing by the standard deviation.

### Statistical analysis

The data analysis was performed with IBM SPSS (v26) to compare both groups. More specifically, two-group comparisons were performed using nonparametric tests due to the nonapproximation to the normal distribution of the quantitative variables, and *p* < 0.05 was set as significant.

For statistical analysis of both groups in fALFF and ReHo, DPABI was used with a voxelwise two-sample t test. As mentioned above, both groups differed significantly in head motion; therefore, Jenkinson’s FD^61^ was included as a covariant in all analyses. Significant differences in the study were reported using the criteria of multiple comparisons with the threshold-free cluster enhancement (TFCE), which reached the best balance between familywise error and test–retest reliability^27^. In total, 10,000 permutations were performed, and the Cluster *p value* was set to *p* < 0.05. An additional threshold with a minimum extent threshold of 30 voxels for ReHo and 10 voxels for fALFF was set to exclude very small clusters, although they passed the strict permutation test with TFCE correction.

Moreover, R Studio (R 4.1.2) was used for the correlations, regression analysis, and visualization matrices. Extraction of the cluster values with DPABI was performed in the clusters where a significant t value was found in the comparisons between DS and controls. Then, they were separately correlated with the cognitive outcome. For this analysis, participants with DSs who did not have KBIT-2 scores were excluded.

### Ethics approval statement

The Bioethical Committee of the Universitat de Barcelona approved the project (03/16/2017).

### Patient consent statement

For minors and the participants with DS, informed consent of the guardians in legal charge of every person with DS was obtained. Informed consent was also acquired from all DS and control participants.

## Results

In Table 1, the participants’ characteristics are shown. As no normality was found in the quantitative variables, the statistical analysis was performed with nonparametric tests. As shown, significant differences were found in head motion, phonological and semantic verbal fluency, and all subtests of KBIT-2.

### fALFF results between groups

**Figure 1:**
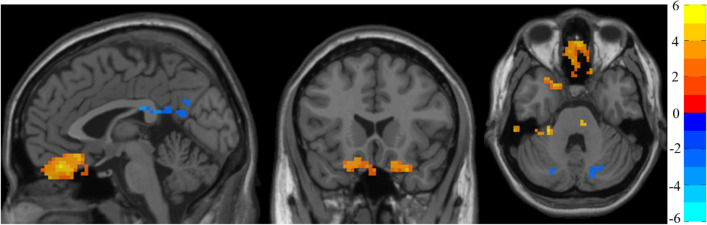
fALFF analysis. Two-sample t test results are presented, corrected by a permutation test with TFCE, p < 0.05. The area in blue represents a significantly decreased ALFF value in DS patients compared with controls; the area in yellow and red represents a significantly increased fALFF value in DS patients compared with controls.

Table 2 shows the significant differences between groups in fALFF with the coordinates of the MNI. Figure 1 shows the graphical representation of the results in fALFF visualized with DPABI^58^.

**Table 2:** Significant between-group differences in fALFF.

Comparison	Area	Number of voxels	*t* (peak)	Peak MNI coordinates (mm)			AAL peak region
DS > C	Cluster1: Frontal and temporal lobe	636	6.36	− 18	12	− 27	~ Temporal_Pole_Sup_L
	Cluster2: Left cerebellum anterior lobe	10	6.19	− 27	− 33	− 33	Cerebellum_4_5_L
	Cluster3: Left inferior temporal lobe	13	5.39	− 60	− 30	− 33	~ Temporal_Inf_L
	Cluster4: Left frontal lobe	40	5.09	− 15	63	− 9	Frontal_Sup_Orb_L
DS < C	Cluster5: Left parietal and occipital lobe	41	− 5.97	− 42	− 75	33	Occipital_Mid_L
	Cluster6: Left limbic lobe	25	− 5.71	0	− 39	21	~ Cingulum_Post_R
	Cluster7: right cerebellum posterior lobe	215	− 5.61	30	− 66	− 36	Cerebellum_Crus_1_R
	Cluster8: left cerebellum posterior lobe	120	− 5.17	− 39	− 66	− 45	Cerebellum_Crus2_L
	Cluster9: Left limbic lobe	23	− 4.77	0	− 63	30	Precuneus_L

C: Controls; MNI: Montreal Neurological Institute; ~ : approximately, AAL atlas area closer to the t peak.

Compared with matched controls, on the one hand, DS showed significantly increased fALFF in the frontal and temporal lobes and the left cerebellum anterior lobe. On the other hand, the DS showed decreased activity in some parts of the left parietal, occipital and limbic lobes and in the left cerebellum posterior lobe.

### ReHo results between groups

**Figure 2:**
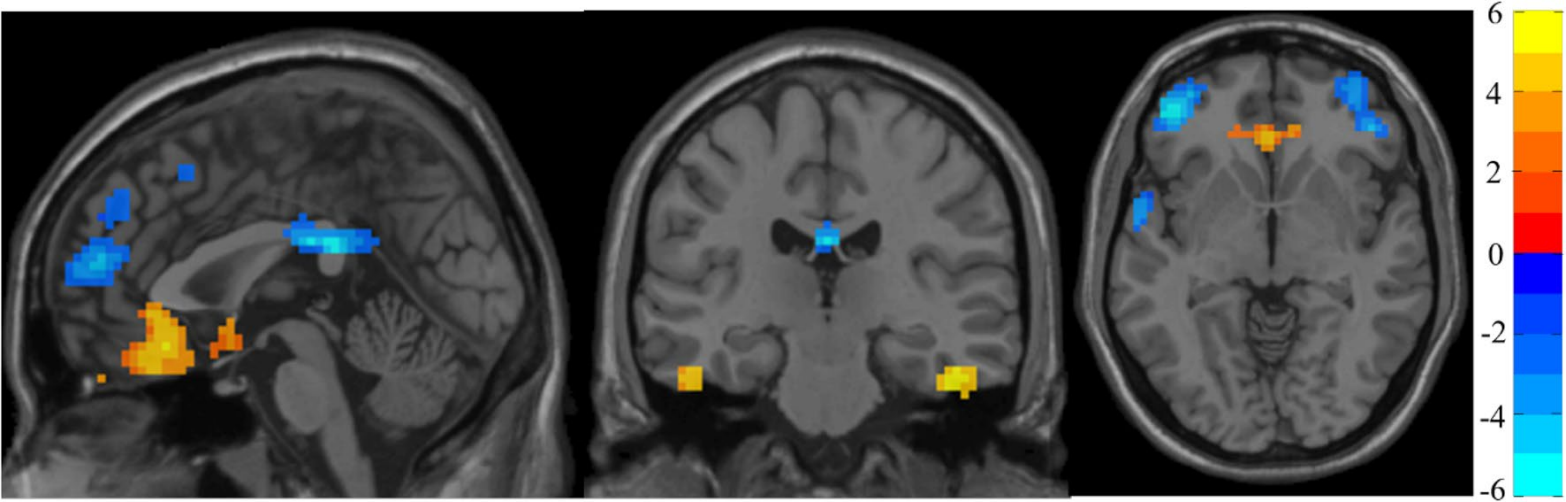
ReHo analysis. Two-sample t test results corrected by TFCE are presented. The area in blue represents a significantly decreased ReHo value in DS patients compared with controls; the area in yellow and red represents a significantly increased ReHo value in DS patients compared with controls.

Table 3 shows the significant differences between groups in ReHo with MNI coordinates. Figure 2 shows the graphical representation of the ReHo results visualized with DPABI^58^.

**Table 3:** Significant between-group differences in ReHo maps.

Comparison	Area	Number of voxels	*t* (peak)	Peak MNI coordinates (mm)			AAL peak region
DS > C	Cluster1: Right inferior temporal lobe	43	5.84	48	− 27	− 27	Temporal_Inf_R
	Cluster2: Left frontal lobe and left rectus	746	5.75	− 3	30	− 15	Rectus_L
	Cluster3: Left inferior temporal lobe	40	5.16	− 42	− 30	− 21	Fusiform_R
DS < C	Cluster4: Frontal lobe	1947	− 7.42	− 45	45	0	Frontal_Mid_Orb_L
	Cluster5: Left limbic lobe	92	− 6.73	0	− 30	24	~ Cingulum_Post_L

*C:* Controls*; MNI:* Montreal Neurological Institute.; ~ : approximately, AAL atlas area closer to the t peak.

Compared with matched controls, on the one hand, people with DS showed significantly increased ReHo in some parts of the left frontal lobe and inferior temporal lobe. On the other hand, controls showed significantly increased activity in parts of the frontal lobe and right limbic lobe.

### Correlations and regression analysis

As large differences were found between the control and DS groups through both the ReHo and fALFF techniques, the possible relationship between the significant clusters and cognitive scores/measures was analysed via a correlation test.

**Figure 3:**
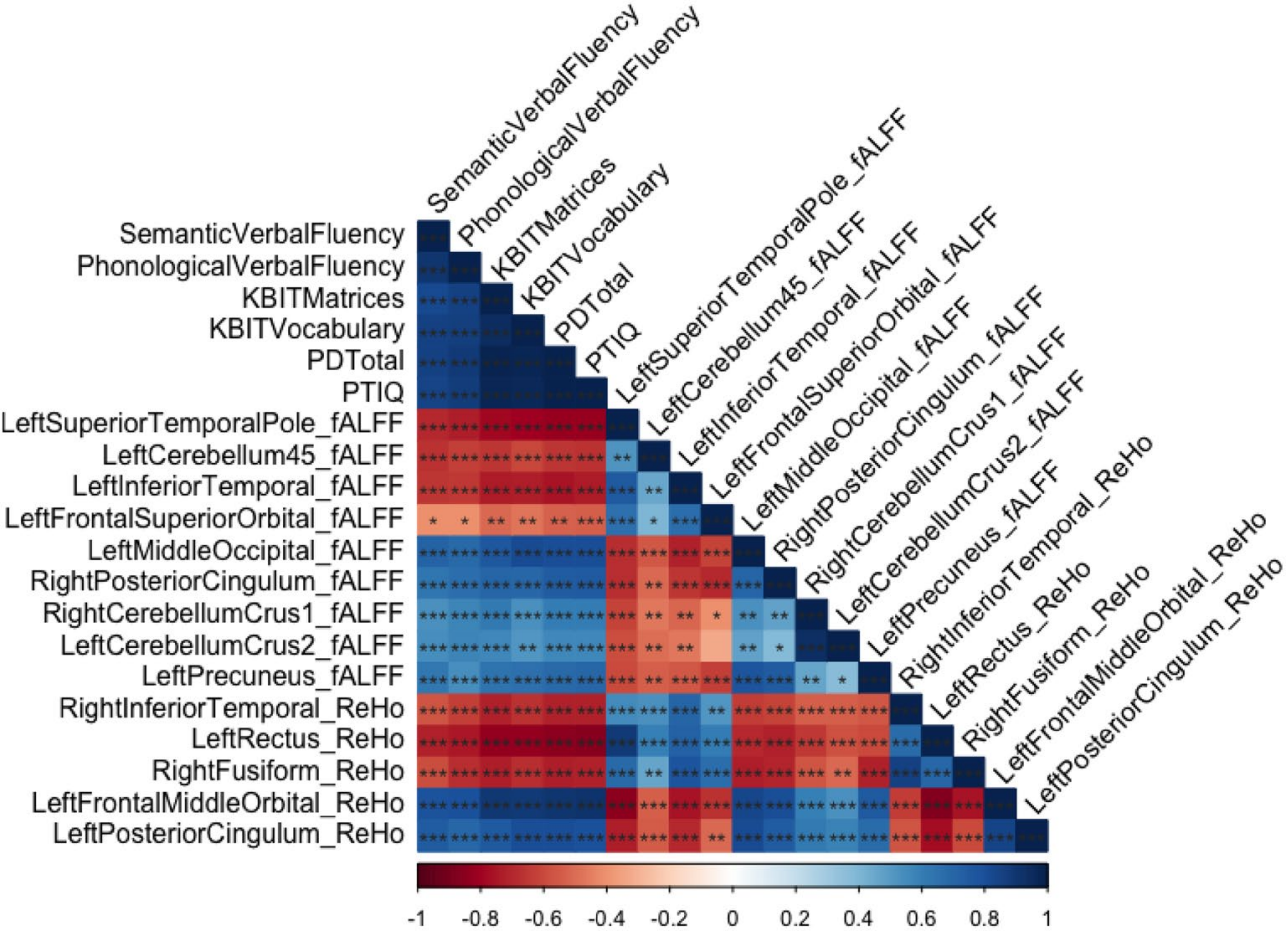
Correlation matrix regarding the cognitive outcome and significant clusters in fALFF and ReHo.

Figure 3 represents a correlation matrix between the different significant clusters in fALFF and ReHo and cognitive scores. As expected, a high correlation was found between all cognitive measures and between the cluster signals. More interestingly, Fig. 3 highlights the high correlations between the cognitive measures and the cluster values.

Regarding fALFF, at a *p* < 0.001 significance level, all clusters correlated with most of the cognitive measures. The first four clusters of fALFF correlated negatively with cognitive scores, and the five final clusters were positively correlated with cognitive scores. Moreover, the directions of the correlations were consistent with the sign of the comparison, indicating that, when DS had increased activity in a cluster, the signal in that cluster was negatively correlated with the cognitive outcome. Similarly, when the controls had increased activity in a cluster, then the signal in that cluster was positively correlated with the cognitive outcome.

Regarding ReHo measures, similar to fALFF at a *p* < 0.001 significance level, all clusters correlated with most of the cognitive measures. The first three clusters of ReHo correlated negatively with the cognitive scores, and the two final clusters were positively correlated with the cognitive scores. The directions of the correlations were consistent with the sign of the comparison, indicating that, when DS had increased activity in a cluster, the signal in that cluster was negatively correlated with the cognitive outcome. Similarly, when the controls had increased activity in a cluster, the signal in that cluster was positively correlated with the cognitive outcome.

As the correlations in both the fALFF and ReHo clusters were high, a regression analysis was performed for every cognitive measure. It is important to highlight that the total measures of IQ in KBIT-2 were excluded from the regression models because the two groups were easy to distinguish and were not logical. Therefore, only the cognitive measures with sufficient variability, such as phonological and semantic verbal fluency, matrices, and vocabulary, were used as criterion variables. As a consequence, four step-wise regression models were adjusted including as predictor variables the 14 clusters obtained in the fALFF and ReHo analyses.

In Table 4, the regression models predicting cognitive scores are presented. They all met the conditions (no error autocorrelation, linearity, normality, and homoscedasticity of errors tested). All of them had high *R*^2^ values, meaning that a high level of prediction was achieved. All of them were multiple linear regressions, and only some variables were included as predictors, including in the KBIT-2 matricces and vocabulary regression four variables, and two of them in the prediction of phonological and semantic verbal fluency. It is important to highlight that the predictor variables of semantic and phonological fluency regression model were the same.

**Table 4:** Parameter estimation (β) of the best stepwise linear model for each significant cluster in fALFF. They all had a *p* < 0.001 in the model (df = 1; 33) and a *p* > 0.05 in Anderson’s Darling test of normality, the Ramsey Regression Equation Specification Error (RESET) test, Durbin Watson’s test, and the Breusch–Pagan test.

Signal	KBIT-2 matrices	KBIT-2 vocabulary	Phonological verbal fluency	Semantic verbal fluency
	*F* = 53.65 *R*^*2 *^*= *0.86 *AIC* = 221.51	*F* = 43.17 *R*^*2*^ = 0.83 *AIC* = 269.07	*F* = 34.22 *R*^*2 *^*= *0.66 *AIC* = 214.56	*F* = 37.17 *R*^*2 *^*= *0.68 *AIC* = 276.38
Intercept	5.91	13.58	3.59	15.02
Left Cerebellum 4,5 fALFF	− 7.98		− 6.82	− 20.73
Left Superior Temporal Pole fALFF		− 25.32		
Left Frontal Superior Orbital fALFF	5.63	13.92		
Left Middle Occipital fALFF		16.13		
Right Inferior Temporal ReHo	− 6.73			
Left Frontal Middle Orbital ReHo	25.17	27.687	12.14	28.32

*F* ANOVA, *AIC* Akaike information criterion.

Supplementary Figs. 1 and 2 show the scatter plots for the prediction of KBIT-2 Matrices and Vocabulary, as well as verbal fluency.

## Discussion

There is a scarcity of neuroimaging studies involving people with DS, especially in rs-*f*MRI techniques for detecting regional signal changes in spontaneous activity. Therefore, this study aimed to study the whole-brain resting state using fALFF and ReHo strategies to find differences in spontaneous brain activity among young people with DS and controls. Moreover, this study aimed to correlate the results of the differences between controls and DSs with cognitive outcomes.

Regarding fALFF analysis, the results showed significant differences in these frequencies in the whole brain between both compared groups. The areas that showed increased activity in DS included some parts of the frontal and temporal lobes and the left cerebellum anterior lobe. The areas that showed decreased activity in DS compared with controls were regions of the left parietal and occipital lobe, the left limbic lobe and the left cerebellum posterior lobe.

In relation to the ReHo analysis, significant differences between both groups were found. The areas that showed increased activity in DS participants compared with controls were certain areas of the left frontal lobe and left rectus, as well as the inferior temporal lobe. The areas with decreased activity in the DS participants were regions of the frontal lobe and the right limbic lobe.

There was high congruence between the areas in which differences were found using both techniques, which is consistent with previous studies^64^ in other populations. Regarding the higher activity in the DS population than in the controls, the congruent regions of both analyses were the frontal and temporal lobes. Compared with the controls, the limbic lobe was decreased in DS participants. The areas that show widespread differences between controls and DS are clearly abnormal in DS, finding increased and decreased activity. Moreover, as no neuropathology of AD was present in our sample, these differences were not due to the clinical features of AD.

As we hypothesized, DS participants showed decreased fALFF and ReHo in the limbic lobe, a region that conforms to the DMN. Rosas et al.^17^ also found differences in connectivity in this region. Moreover, they found an increased level of local connectivity within the frontal lobe in DS, which is consistent with our results showing increased fALFF and ReHo in the frontal lobe in DS compared with controls. Wilson et al.^19^ also found weaker connectivity in the DS group than controls with the DMN seed and several regions, some of which were also reported in our study. More specifically, the precuneus, reported in our research as having decreased fALFF in the DS group than in the controls, and part of the cerebellum also showed significant results in our study.

Moreover, increased fALFF and ReHo have also been found in the frontal and temporal lobes. Both lobes are relevant for executive and language functions and memory^53,54^, functions highly altered in DS^33,65,66^. This increase in spontaneous activity, despite being in a rs-*f*MRI paradigm, could be a mechanism of compensation for their disrupted networks and functions, as has been suggested in other pathologies that present cognitive impairments^67^ or visual disabilities^68^.

Structural brain abnormalities have been found in DS compared with controls, and the areas in which they are found are consistent with our study. For instance, Beacher et al.^8^ and Newton^13^ found a specific volume reduction in the frontal, temporal, and parietal lobes and the cerebellum, areas also appearing abnormal in our study. Beacher et al.^8^ demonstrated that these areas show more significant age-related reduction than the general population. Additionally, decreased white matter integrity has been found in the frontal tracts, and correlations with cognitive dysfunction have been demonstrated^9,10^. In this study, differences in fALFF and ReHo involving white matter and frontal areas were found. Therefore, white matter appears to play an important role in the neuropathology of people with DS. Cerebellar alterations have been reported in this population. An investigation in foetuses with DS found that the cerebellum had an immature pattern, a reduced volume and notably fewer cells in all cerebellar layers^69^. Other authors have reported similar results^70–72^. Recently, the importance of cerebrocerebellar networks in the clinical manifestations of DS has been highlighted. More specifically, researchers found hypoplasia of cerebellar afferent systems in DS using DTI-driven tensor-based morphometry^73^. In our study, differences in fALFF were found, but interestingly, increased fALFF was found in the anterior region of the cerebellum, and decreased fALFF was found in the posterior region of the cerebellum. Rosas et al.^17^ found an anterior–posterior dissociation in this population in the DMN, and perhaps this could also be widespread in the cerebellum.

Regarding grey matter, Anderson et al.^29^ found that DS presented higher levels of synchrony between most grey matter regions. DS also exhibits higher fALFF and ReHo in the fusiform gyrus. This abnormality may be explained because this region plays a crucial role in visual recognition memory, a function that is impaired in children and adults with DS. Guidi et al.^74^ found reduced thickness in this region in foetuses with DS compared with controls.

Despite the young sample of DS used in this study, it has been demonstrated that the DS population without dementia exhibits β-amyloid (Aβ) burden that can begin in the late teens^24,25^. Orbitofrontal regions have proven to be areas affected by this early deposition in DS presenting MCI but also in cognitive stable DS^75,76^. The abnormalities found in the orbitofrontal lobe (increased fALFF in DS) could be a prelude of this deposition in our sample, but more studies are needed to demonstrate this association by linking the amyloid deposition with fALFF values. To date, this association has only been demonstrated with ReHo^49^, with higher values of ReHo indicating amyloid deposition.

These results should also be compared with AD studies using fALFF and ReHo. Yang et al.^48^ found decreased fALFF in the right precuneus in AD and MCI participants compared with controls. In our study, this area also presented decreased activity in fALFF in DS. Cha et al.^77^ also found significant differences in the precuneus between AD patients and controls, finding increased values of fALFF in controls, as in our study. As mentioned before, although the results with AD could be consistent in some areas with the results of this study, the younger population used in this study can guarantee that the symptomatology of AD is not present in the sample. However, neuropathology in the brain can begin years before clinical symptoms are evident, and amyloid deposition is already present in young DS^75,76^. Moreover, differences in brain structure development can increase vulnerability to AD in DS^78^. Because of the high incidence of AD in DS, the DS population provides an extraordinary opportunity for understanding the temporal progression of AD and the different facets that contribute to the age of dementia onset and for applying this knowledge to the general population^4^.

Interestingly, there was a high correlation between all of the significant clusters in fALFF and ReHO and cognitive measures. We can affirm that the differences between DS and controls in fALFF and ReHo measures have a significant relationship with the cognitive profile of both groups and are linked to verbal and nonverbal intelligence, as measured with KBIT-2. Moreover, these correlation analyses were consistent with the sign of the relationship found between DS and the controls. This study demonstrates that the differences in congruity and low-frequency fluctuations seen between the controls and DS depend on its cognitive profile because high correlations have been found between the signal in fALFF and ReHo in the different respective clusters of cognitive outcomes. Yang et al.^48^ found a relationship between fALFF and cognitive outcome in a sample with an AD spectrum. They found significant correlations between the whole sample (including controls) and neuropsychological outcomes. As high correlations were found between cognitive measures and cluster activity through both analyses, a regression was performed using only the variables that had high variability in their distribution, such as KBIT-2 Vocabulary and Matrices raw scores as well as semantic and phonological verbal fluency. It is important to remark that KBIT-2 total outcomes are not presented as criterion variables in any model because the dispersion found in the variables clearly allows identification of both types of populations. As predicted, the results found in DS and the controls in those variables were truly different, and both groups were located in the opposed tail of the distribution. Notably, the differences found in the clusters of fALFF and ReHo can be explained by some cognitive variables, such as verbal- and nonverbal-related measures. All of the regression models were highly predictable, with high *R*^2^ values, and all of the models include some of the cluster's signals detected by fALFF and ReHo analyses. Studies using task procedures in *f*MRI have demonstrated the engagement of the left cerebellum in nonverbal tasks^79^. Studies have also highlighted the role of the left middle orbital superior frontal gyrus in phonological processing accuracy in children with dyslexia^80^. Finally, regarding the engagement of the left hemisphere in vocabulary, studies have demonstrated a language lateralization to the left part of the brain in healthy adult participants^81^.

Taken together, significant differences in fALFF and ReHo were found between DS and controls. Some of the differences encountered could be related to the neuropsychological profile of the DS (engagement of the frontal and temporal lobes as hubs for executive and language functions). We suggest that this increased fALFF could be a compensatory mechanism, as has been suggested in other studies with participants who present cognitive impairments^67^ or visual disabilities^68^ or in healthy ageing^82^. Other differences could be due to the already demonstrated amyloid deposition that takes place within the ages of our sample in cognitive stable DS. Some of the results found are also linked with the structural abnormalities already found in this population in the frontal, temporal, parietal and cerebellar lobes. Moreover, abnormalities have also been found in the DMN, a network that has already been demonstrated to be disrupted in DS.

Finally, as both techniques used in this study are data-driven, it is not necessary to have a prior hypothesis of the seed regions in the analysis^83^. Specifically, in the DS population, as there is a scarcity of studies in rs-*f*MRI, both techniques are valuable tools for studying this population without knowing the specific underlying pathology. Both techniques have localized brain areas that are different in both populations. The specific regions that have shown differences between both groups could be targeted brain regions for future lines of research, using them as seeds to explore the FC to other regions in the brain. Moreover, this exploratory study demonstrates the regions that are abnormal in this population and is the first step to disentangle the pathological functions and connectivity of the DS brain due to lifelong abnormal development. This study is also important for understanding ageing in DS and the incidence of AD pathology in this population.

The correlations and regression analysis performed in this study demonstrate that differences in both groups depend highly on their cognitive profile, and the level of prediction for the cognitive evaluation is high.

This study is not exempt from some limitations. In this sense, the difficulties in recruiting samples in this particular population and, of course, the high degree of movement during scanning in the DS population limited the study's sample size. However, it is important to highlight that this study’s sample size is in accordance with the typical sample size used in neuroimaging^28^. Therefore, more studies using FC and other methodological approaches, such as graph analysis or ReHo and fALFF, in this population are needed, targeting the areas found to be disrupted in this study as seeds. The lack of a replication dataset may have also limited the results. Finally, motion, even if well controlled, could have affected the results.

There are also some strengths of the study that are worth mentioning. To our knowledge, this is the first study to explore regional spontaneous brain activity in a population with DS compared to controls. Moreover, the high control of the age of the study is valuable because of the early onset of AD in this population. This study allows us to affirm that the differences found between the two populations are not because of the clinical symptomatology of AD. Finally, a highly restricted correction for multiple comparisons was performed in this analysis, and multiple corrections for movement were performed. The results had a large effect size; thus, we could affirm significant differences in regional spontaneous brain activity between controls and DS using fALFF and ReHo. Moreover, this study suggests the possibility of using fALFF and ReHo as biomarkers of cognitive function, which is highly important given the difficulties in cognitively evaluating this population and assessing dementia^6^. Both techniques have been suggested to be potential biomarkers owing to their high test–retest reliability^43,44^. More research is needed, however, to demonstrate its utility.

## Supplementary Information

Supplementary Figures.

## Data Availability

The data that support the findings of this study are available on request from the corresponding author. The data are not publicly available due to privacy or ethical restrictions.
