# Supplementary material for: Altered spontaneous brain activity in Down syndrome and its relation with cognitive outcome
Source: Sci Rep. 2022 Sep 14;12:15410. doi: 10.1038/s41598-022-19627-1 (PMC9474876; doi:10.1038/s41598-022-19627-1)

**Supplementary Information**

**Supplementary Figure 1.** Scatter plot and regression for KBIT Matrices and Vocabulary. In the left, Matrices raw score is predicted, and in the right, Vocabulary raw score is predicted.


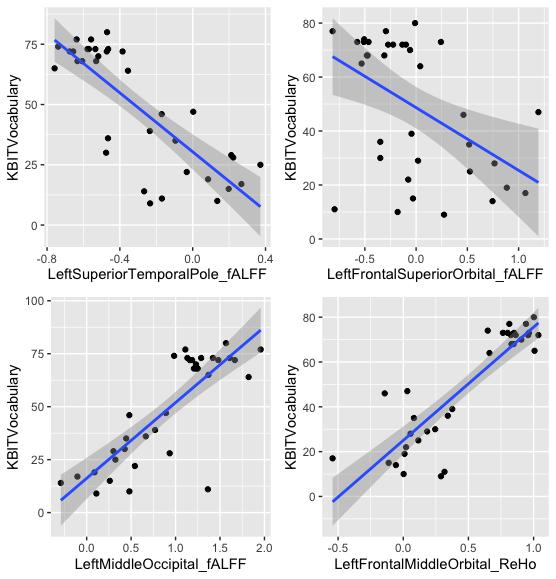

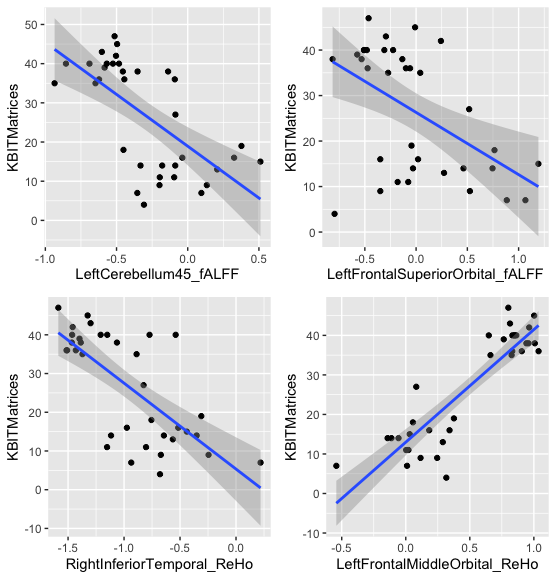


**Supplementary Figure 2.** Scatter plot and regression for verbal fluency. A represents the prediction of phonological verbal fluency and B represents the prediction of semantic verbal fluency.


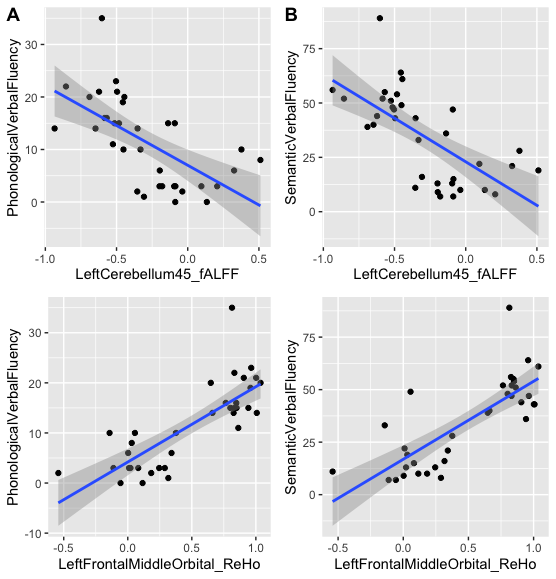

Supplement: Supplementary file 1 — Supplementary Figures. [file 41598_2022_19627_MOESM1_ESM.docx]
